# *Helicobacter pylori* vacA genes associated with gastric diseases in Trinidad and Tobago

**DOI:** 10.1016/j.ijregi.2024.100498

**Published:** 2024-11-22

**Authors:** Chandrashekhar Unakal, Lemar Wayne Blake, Gerard Farfan, Angel Justiz-Vaillant, Bijay Raj Pandit, Patrick Eberechi Akpaka

**Affiliations:** 1 Faculty of Medical Sciences, The University of the West Indies, St. Augustine, Trinidad and Tobago; 2 The Endoscopy Centre, Woodbrook, Port of Spain, Trinidad and Tobago

**Keywords:** *Helicobacter pylori*, Vacuolating cytotoxin A (*vac*A), Gastric disorders, Polymerase chain reaction, Trinidad and Tobago

## Abstract

**Objectives:**

To delineate and understand the genetic variations among *Helicobacter pylori* strains from Trinidad and Tobago associated with gastric diseases.

**Methods:**

One hundred (n = 100) patients who routinely presented with clinical features suggestive of peptic disease were enrolled in the study and underwent gastroscopy procedures. Biopsy specimens were analyzed using serological and molecular methods. Amplification of the variable vacuolating cytotoxin gene A (*vac*A), urease C (*ure*C), and 16S ribosomal RNA regions was performed by polymerase chain reaction; and then followed by sequencing and alignment. Phylogenetic analysis and DNA fragment alignment were carried out using DNA MEGA 7 version 7.

**Results:**

Overall, 70 of the patients tested positive for *H. pylori* infection using an enzyme immune assay. All were 16S ribosomal RNA positive, with 8.6% (6/70) testing positive for S1 and 91.4% (64/70) for S2. The most prevalent *vac*A allelic combination was S2M1, with 87.1% (61/70) cases. All S1M1 strains were *ure*C-positive, while S1M2 strains were *ure*C-negative.

**Conclusions:**

*H. pylori* strain variations are seen in the Trinidad and Tobago isolates and are most closely related to the ones from the USA, India, and Indonesia. However, the factors contributing to the emergence of S2/M1 being more prevalent will need to be further investigated.

## Highlights

• A high incidence of *Helicobacter pylori* exists among patients with peptic ulcers in Trinidad and Tobago.
• S2m1 is the most prevalent *H. pylori vac*A gene type in Trinidad and Tobago.
• The results of polymerase chain reaction were excellent for the variable *vac*A and 16S ribosomal RNA regions of *H. pylori*.
• Trinidad and Tobago *H. pylori* strains are closely related to USA, India, and Indonesia.
• Gastric carcinoma and gastric-mucosa-associated lymphoid tissue caused by *H. pylori* were nonexistent in the country.

## Introduction

*Helicobacter pylori* is a spiral microaerophilic Gram-negative organism that was discovered in 1983 [1], and it is one of the most common bacteria infecting approximately half of the world's population in several regions [2]. The bacterium can cause a variety of gastrointestinal diseases because of its distinct infection, colonization processes, and disease mechanisms that are determined by abilities to produce several enzymes and toxins [3]. This organism colonizes the stomach of approximately 50% of the worldwide population [3,4]; and it is responsible for disorders such as gastritis, gastric and duodenal ulcers, gastric mucosa-associated lymphoid tissue (MALT) lymphoma, and gastric adenocarcinoma [4,5]. Gastric cancer caused by *H. pylori* is found in only 0.1% to 1% of infected people in the Western world, where infection is said to be about 30% [4,5]. Counties such as South Korea, China, Japan, and India have a prevalence between 60-80% and are known to have greater gastric cancer cases [6].

*H. pylori* can be easily identified using specific primers for conserved regions (16s ribosomal RNA [16S rRNA]) [7]. Strains that colonize the human intestine have been shown to possess urease (*ure*A, B), and *ure*C which is vital for colonization and is a potent immunogen [8]. The ability of *H. pylori* to cause severe infection is due to risk factors, such as gene expression and allelic variations, especially of the vacuolating cytotoxin gene A (*vac*A) and the cytotoxin-related gene A (*cag*A) [9]. These toxins (*cag*A and *vac*A) are the main virulence factors of *H. pylori*, contributing to its steep global infection rate [10]. *Vac*A exhibits variable regions, with alleles including type m1 (subtype a), m2 (subtype a or b), s1 (subtype a, b, and c), or s2 [11]. The *vac*A gene is virtually present in all *H. pylori* strains and has at least two variable regions, the signal (s) region -encoding the signal peptide, and the middle (m) region. The s-region has two major s-region types, s1(with three subtypes - s1a, s1b, s1c) and s2. The m-region has two allelic types - m1 and m2; the m1 type has subtypes m1a and m1b [9,11]. The variable regions of the *vac*A gene; alleles of these include type m1 (subtype a) m2 (subtype a or b), s1 (subtype a, b, and c) or s2; and the *vac*A s1 strain is implicated in 90% of duodenal ulcer cases [[11], [12], [13]].

This *vac*A highly produced by *H. pylori* or its correlation with gastric diseases has not been investigated in Trinidad and Tobago (TT). This report hence focused on elucidating the diversity of the *vac*A gene of *H. pylori*. This will not only add to the plethora of data published by others, but it will also delineate suitable approaches to clinical diagnosis, laboratory identification, and treatment of *H. pylori* infections.

## Materials and methods

In this cross-sectional study, 100 adult patients (ages >18 to 65) with a history suggestive of gastric disease and attending the Endoscopy Center clinic were subjected to gastroscopy, over a period of 7 months.

### Inclusion and exclusion criteria

Only patients who had no prior history of chronic severe medical illness, who provided written consent, and who had no history of previous *H. pylori* eradication, were experiencing hypochlorhydria, epigastric pain, vomiting, indigestion, and radiation were included in the study. Those patients who were on antibiotics and or proton pump inhibitors one or more weeks prior to endoscopic procedure, or had contraindications of gastric biopsy, were excluded from the investigation.

The gastric biopsy specimens were collected consecutively from each patient and were analyzed according to standard laboratory procedures at the Microbiology Laboratory, Unit of Pathology and Microbiology, Department of Pathology/Microbiology & Pharmacology, The University of the West Indies, St. Augustine. A total of four gastric biopsy specimens were collected from each patient. Samples were collected in sterile universal containers (10 mls 0.9% saline added) and transported to the laboratory immediately for immunoassay (EIA), culture, biochemical and molecular analysis.

DNA extraction: Bacterial DNA was extracted from gastric biopsy tissues using QIAamp® DNA Mini Kit (QIAGEN, Hilden, Germany) following the manufacturer's instructions. The extracted DNA was quantified using the Thermo Scientific™ NanoDrop™ OneC Microvolume UV-Vis Spectrophotometer (Waltham, Massachusetts, USA). The quantification process was performed according to the manufacturer's instructions. DNA with purity at absorbance value (260/280) = 1.7-2.0.

*Vac*A, *Ure*C, and 16s gene analysis: For DNA Amplification, 5 µl of the resultant DNA extractions were added to separate polymerase chain reaction (PCR) tubes containing 20 µl reaction mixture which contained: 5 µl of (cell gene technologies) taq polymerase, 0.5 µl of each primer that was used for that reaction 14 µl of PCR water. This gave a total of 25 µl per reaction. The PCR assay was performed as described previously in the literature with some slight modifications [13]. Master Mix contains reaction buffer (pH 8.5), bacterially derived Taq DNA polymerase 0.63 units, 200 µM dATP, 200 µM dGTP, 20 µM dCTP, 200 µM dTTP and 1.5 mM MgCl2. PCR analysis for the 16s rRNA and *vac*A was carried out as shown in the studies referenced (Table 1).

**Table 1:** PCR conditions and primers used for *Helicobacter pylori*

Gene	Primer sequence	AT	ET	FET	Size	Ref
16srRNA	Forward	58^o^C	72^o^C	72^o^C	109	[7,13]
	5’-CTGGAGAGACTAAGCCCTCC-3′)	1min	1min	5min		
	Reverse					
	5′-ATTACTGACGCTGATTGTGC-3′)					
*vac*A s1/s2	Forward	52^o^C	72^o^C	72^o^C	259	[12,13]
	5’-ATGGAAATACAACAAACACAC-3’	45sec	45sec	5min	286	
	Reverse					
	5’-CTGCTTGAATGCGCCAAAC-3’					
m1	Forward					
	‘5-GGTCAAAATGCGGTCATGG-3’					
	Reverse					
	‘5-CCATTGGTACCTGTAGAAAC-3’					
m2	Forward					
	‘5-GGAGCCCCAGGAAACATTG-3’					
	Reverse					
	‘5-CATAACTAGCGCCTTGCAC-3’					
*ure*C	Forward	64^o^C	72^o^C	72^o^C	294	[7,13]
	5’-AAGCTTTTAGGGGTGTTA	1min	1min	7min		
	GGGGTTT-3’					
	Reverse					
	5’-AAGCTTACTTTCTAAC					
	ACTAACGC-3’					

AT = Annealing temperature; ET = Extension temperature; FET = Final Extension temperature; Size = base pairs size; Ref = Reference.

Molecular detection of *H. pylori* and its virulence genes: Fifty nanograms of DNA were utilized to detect the presence of *H. pylori* using 16S rRNA-specific primers, as described previously in literature with some slight modifications [7]. Following the amplification of 16S rRNA, positive samples were selected for the detection of the virulence genes *cag*A and *vac*A using specific primers based on what was reported in the literature [13]. PCR was performed in a total volume of 12.5 µL containing twice master mix with standard buffer (NEB Inc., Ipswich, MA) and 0.2 µM of forward and reverse primers. The primer sequences and annealing temperatures are described in Table 1 below. The PCR products were run on 1.2% agarose gel and visualized under an ultraviolet (UV) light using the GelDoc EZ Gel Imaging System (Bio-Rad, Hercules, CA). The ATCC 43504 *H. pylori* strain was employed as a control in all tests.

DNA sequencing and phylogenetic analysis: Amplified DNA products from the positive samples were sent to Macrogen Sequencing Company, South Korea for sequencing of the gene fragment of interest. Phylogenetic analysis and DNA fragment alignment were carried out using DNA MEGA 6 software, according to the program instructions.

### Statistical analysis

The results were entered into the SPSS version 29 software (IBM Armonk, NY, USA) and statistical analysis was carried out where necessary by using the paired and one-sample *t*-test. Participants were categorized as positive or negative for *H. pylori* and its associated genes based on identification from specimens. An analysis that had a *P*-value of ≤0.05 was regarded as statistically significant.

## Results

The results of the EIA and 16S rRNA confirmed a high prevalence of 70% (70/100; *P* <0.0001) of *H. pylori* in patients recruited for this study, and this comprised 57.1% (40/70) men and 42.9% (30/70; *P* <0.04) women. The gender distribution of the *H. pylori* disease severity reveals that more men 58.8% (40/68) suffered from gastric abnormalities when compared to women 32.4% (22/68), and eight patients were positive for *H. pylori* but had no gastrointestinal disturbances of any sort. There were no cases of G-MALT or gastric carcinoma encountered among the 70 positive patients.

As depicted in Table 2, isolates from 70 patients were EIA and 16S rRNA positive, and the distribution of the variable *vac*A and its alleles were s1, 8.6% (6/70) and s2, 91.4% (64/70) positive, respectively. The s2m1 was the most frequent *vac*A allelic combination accounting for 62.5% (40/64 cases), followed by s1m1 7.8% (5/64 cases), and s2m2, 29.7% (19/64 cases). However, all s1m1 (n = 5) were *ure*C-positive and s1m2 were *ure*C-negative, respectively.

**Table 2:** The gene relationship between patient disease severity, age, gender, and *Helicobacter pylori vac*A allele status (n = 70).

Feature	EIA+	16S ribosomal RNA *vac*A	*vac*A	*vac*A	*vac*A	*vac*A	Total (%)
			s1/m1	s1/m2	s2/m1	s2/m2	
Dx severity							
Normal	6	6	1	0	5	0	6(8.5)
Mild gas	52	52	2	0	47	3	3
Mod gas	10	10	1	1	8	0	10(14.3)
Severe gas	2	2	1	0	1	0	2(2.9)
Gender							
Male	40	40	3	0	17	1	40(57.1)
Female	30	30	2	0	23	2	30(42.9)
Age							
18-19	0	0	0	0	0	0	0
20-29	4	4	0	0	4	0	4(5.7)
30-39	6	6	0	0	4	2	6(8.5)
40-49	13	13	3	0	10	0	13(18.6)
50-59	20	20	2	0	15	3	20(28.6)
60+	27	27	4	0	23	0	27(38.6)
Total	70	70	9	0	56	5	

Dx severity, disease status of the patient; G-MALT, gastric mucosa-associated lymphoid tissue; GC, gastric carcinoma; Mild Gas, mild gastritis; Mod Gas, moderate gastritis; Severe Gas, severe gastritis.

More than half (74.3% [52/70]) of the patients who tested positive for *H. pylori* had mild gastritis, and most of this 67.1% (47/70) had *vac*A s2m2. The age distribution revealed that the most prevalent age group was the 60-65 with 38.6% (27/70), followed by the 50-59 age group with 28.6% (20/70). The least occurred among the age group 20-29 with a small frequency of 5.7% (4/70). No cases were seen among those less than 20 years of age (Table 2).

**Figure 1:**
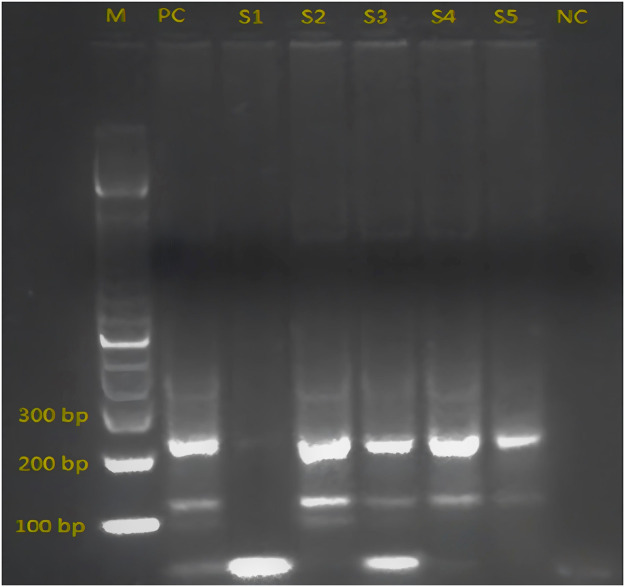
The image illustrates 16S ribosomal RNA via HP1 and 2 primers, from patient isolates. HP, heterochromatin protein; M, DNA 100 bp ladder; NC, negative control; PC, positive control 109 bp; S1-S5, samples.

**Figure 2:**
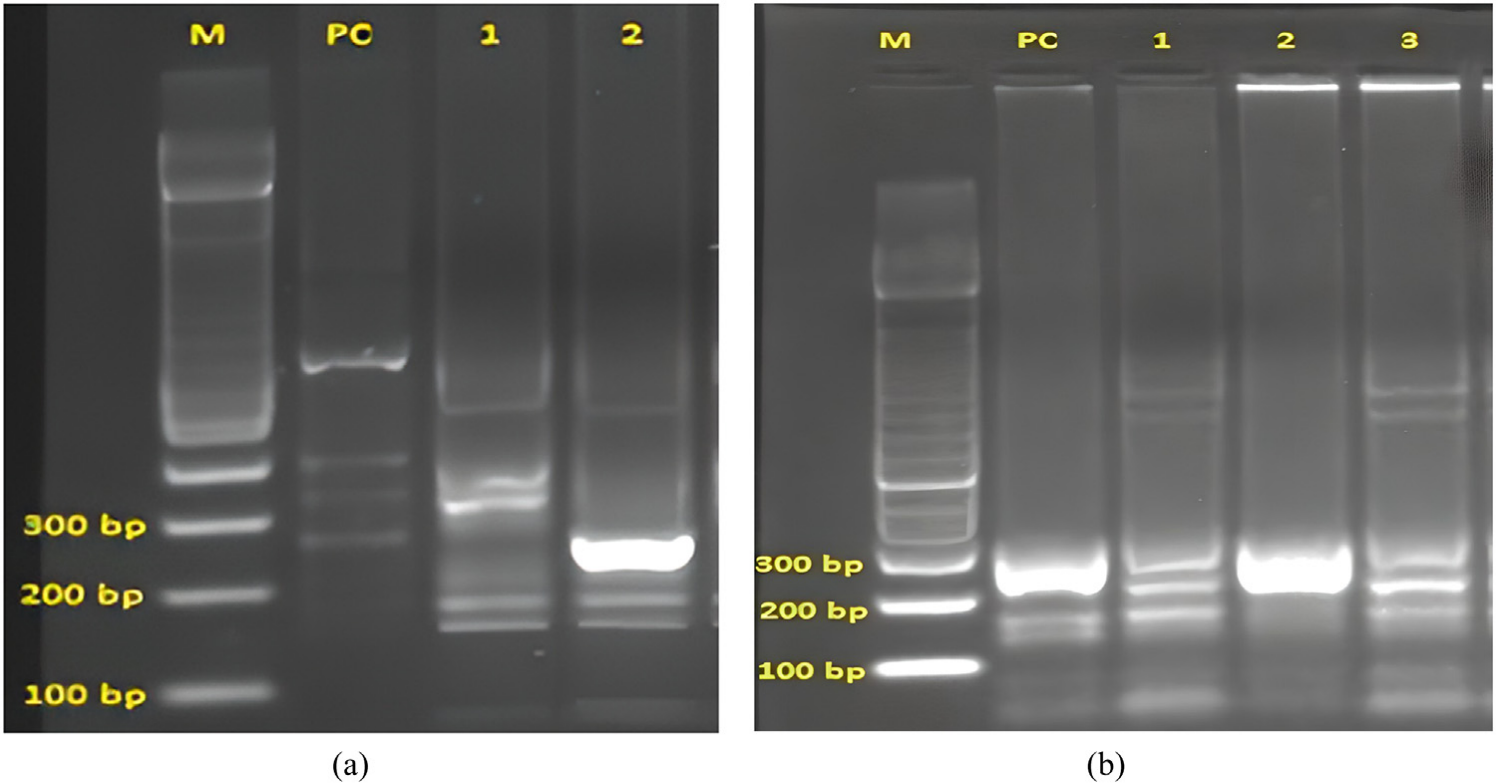
(a and b). The two images show the presence of *vac*A s1\s2 and m1 genes, from Trinidad and Tobago patient isolates. Figure 2a: M, DNA 100 bp ladder, PC, positive control (s2, 289 bp), 1, 2(s1, 250 bp); and Figure 2b: M, DNA 100 bp ladder, PC, positive control (290 bp), 1-3 (*vac*A m1 genes).

**Figure 3:**
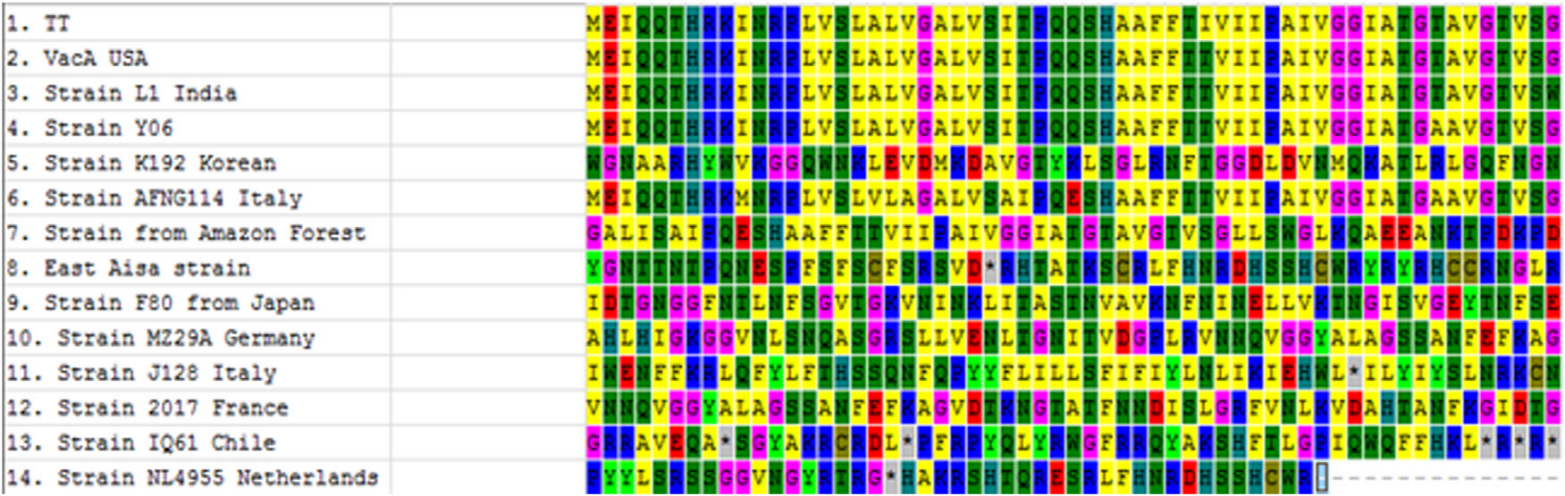
The *vac*A s1 partial amino acid alignment for published strains (NCBI database) vs virulent strain from TT-GenBank KY360307. The amino acid alignment a highly problematic/virulent strain was selected and used for comparison with published strains from the NCBI database. NCBI, National Center for Biotechnology Information; TT, Trinidad and Tobago; USA, United States of America; *vac*A, vacuolating cytotoxin gene A.

**Figure 4:**
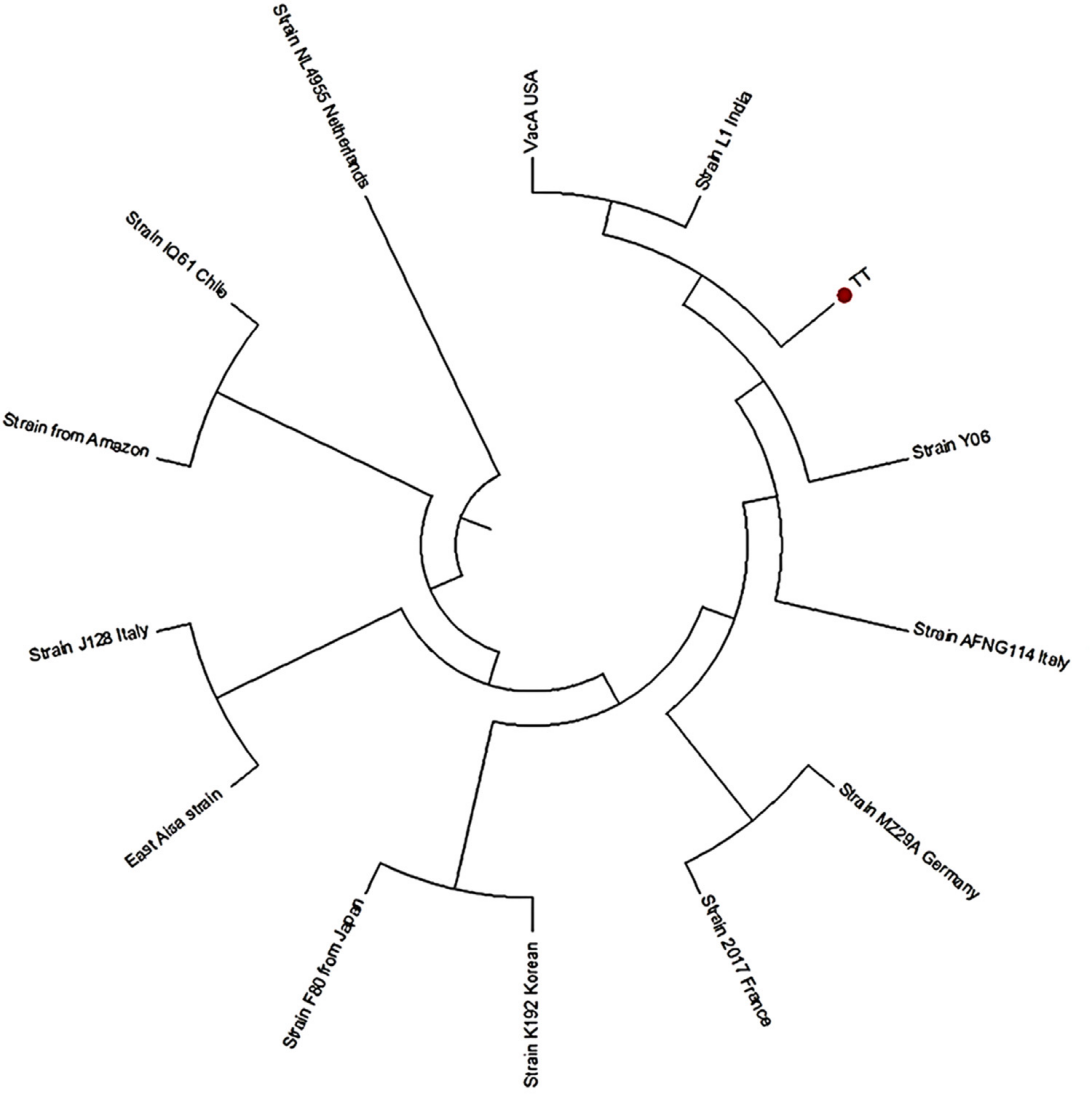
Phylogenetic tree comparing isolates to known *Helicobacter pylori* strains using DNA Mega7 software; GenBank KY360307. TT, Trinidad and Tobago. The tree was inferred using the maximum likelihood method and the Tamura 3-parameter model in MEGA-7 [14].

The results obtained from the amplification of the PCR materials of the isolates are depicted in Figure 1, Figure 2 confirming the results obtained in the Tables above. These figures (Figure 1, Figure 2) illustrate the confirmation of the results obtained when *H. pylori* isolated from the positive patients were analyzed as already tabulated above. Figure 3 depicts the *vac*A s1 partial amino acid alignment for published strains (National Center for Biotechnology Information [NCBI] database) versus virulent strains from TT-GenBank KY360307; Figure 4 shows the use of a phylogenetic tree to group the isolate and compare it to known *H. pylori* strains. However, a highly problematic/virulent strain was selected and used for phylogenetic analysis. This figure shows the strains from TT are most closely related to the USA, India, and the Y06 (Indonesia) strain. Phylogenetic analysis was done using DNA Mega6 software. The NCBI GenBank accession number is KY360307.

Specific amplified products from isolates that were recovered were sequenced and aligned; these showed a 99-100% similarity (not shown). The base pair alignment for a highly problematic strain was selected and used in comparison with published strains from the NCBI database. The result analysis is depicted in Figure 3 which is *vac*A s1 partial amino acid alignment for the published strains (NCBI database) vs virulent strain from TT- GenBank KY360307.

Figure 4 uses a phylogenetic tree to group the isolates and compare it to known *H. pylori* strains. However, a highly problematic/virulent strain was selected and used for phylogenetic analysis. The TT strain is most closely related to the USA, India, and the Y06 (Indonesia) strain. Phylogenetic analysis was done using DNA Mega7 software; GenBank KY360307. The phylogenetic tree comparing isolates to known *H. pylori* strains was inferred using the Maximum Likelihood method and Tamura 3-parameter model in MEGA-7 [14].

## Discussion

In this analysis of delineating the *H. pylori vac*A genes associated with gastric diseases in TT, the report revealed a 70% rate of infection among the cross-section of the population studied in the country. This analysis to the best of our knowledge is the first to be carried out in the country and this rate appears relatively low compared to the infection rates in several African countries where the *H. pylori* rate is even as high as 90% in Libya, Egypt, Nigeria, and other countries [15] when compared to rates from other countries especially in the developing countries [16] where it is noted that over time people's living standards and eating habits are poor. This is not in agreement with data from our country which has been classified as an upper middle-income country by the World Bank where there is a remarkable improvement in people's living standards.

Globally, the prevalence rate of *H. pylori* infection is highest in Africa, Asia, Europe, the Americas, and Oceania, in that order [16,17]. The high rate seen in this study does not in any way reflect what would be the true picture in the country since the study was carried out in a referral center that treats patients with gastrointestinal disorders and there are not too many such centers in the country. The population of the country is about 1.3 million (https://cso.gov.tt/subjects/population-and-vital-statistics/population), this is very much smaller when compared to countries with ≥10 million people.

Most of the people with *H. pylori* infection in our analysis were over the age of 50 years with over 89%, and this is in agreement with what has been reported in the literature as numerous studies have shown that *H. pylori* infection is greatly age-related. As reported by others, *H. pylori* infection rates are very high (95%) in people older than 60 years and low (35%) in adolescents [18]. In this analysis, there is minimal age variation in the infection rate of the participants, and this is not in agreement with reports from Armenia, where the prevalence of *H. pylori* varies; among ages 18-25 years old, it is 13.6%; 26-45 years old: 37.9%; 46-65 years old: 61.4%; over 65 years old: 83.3% [19].

The detection of *vac*A allele s2 was very high (91.5%). The *vac*A has been described to contain three highly variable polymorphic regions known as the signal sequence region (*s*1, s2), the intermediate region (*i*1, *i*2, *i*3), and the mid-region (*m*1, *m*2). It is reported that the *H. pylori* strains with the s1m1 phenotype are more frequently associated with severe disease symptoms unlike strains carrying other combinations of these alleles [20]. Specifically, the *vac*A s1 allele is highly toxigenic and is independent of disease status [21]. In this study, the presence of the *vac*A s2 allele was high at 87.1% (61/70) among the *H. pylori* isolates. This is very much in contrast to findings, especially in Asian countries where *vac*A s1 alleles predominate [11]. Although there was a high carriage of the *vac*A s2, there is a possibility that their presence in patients in TT may not be associated with severe gastric disease abnormalities since it has been demonstrated that patients infected with *H. pylori* carrying the *vac*A s1 allele are at increased risk for developing or progressing to gastric precancerous lesions and gastric carcinoma [22]. However, this is not the case in this study as there is a nonexistence of gastric carcinoma or G-MALT cases among the patients studied in this analysis thus far.

This independent status was demonstrated by the varying degrees of gastritis cases investigated. The *vac*A s2/m1 strain turned out to be the most prevalent in TT and is associated with all the various degrees of *H. pylori* illness. This information is significant for TT and reflects the virulence of the current circulating strain. Previous studies have shown the s1/m1 strain to be the most virulent. However, there seem to be factors contributing to the s1/m1 strain is not the most pathogenic in our region unlike in other regions [11,23]. This will be a cause of further studies of *H. pylori* infections in the country.

In this study, more men were affected, but more women were reported to be the highest carriers of the virulent *vac*A s2/m1 genes. However, in a study by Niknam et al. [24], no existing associations were reported between sex or age with *H. pylori* bacterial infection. Several risk factors (obesity, smoking, lifestyle) of patients experiencing GERD have been described [25], but none of such factors were significantly seen or associated with the patients in our study. This again contrasts with the study by Ríos-Sandoval et al. [26], where all the positive cultures they characterized were isolated from female patients over 60 years of age. This they agreed is a coincidence because of the sample size of their study and could have no further analysis to determine whether there is a relationship.

There was no detection of *ure*C among the *H. pylori* isolates and this is in direct contrast to studies reported elsewhere [27]; where it was found that 100 specimens were rapid urease test positive, of which 60 samples (60%) were PCR positive for *H. pylori ure*C gene. Our study did not perform a rapid urease test and in some studies done by others, the sensitivity and specificity were poor in comparison to molecular analysis [28]. Although many laboratories may be moving away from such methods, in resource-strapped countries like in many developing countries, it is still a very useful testing method to confirm *H. pylori* infection [29].

It is very heartwarming to elucidate that there were no cases of G-MALT or gastric carcinoma among the participants studied in this analysis despite the high infection rate of *H. pylori*. This calls for continuous monitoring and follow-up of these patients as there has been reported a high association between *Helicobacter pylori* infection and gastric carcinoma and G-MALT [30].

## Conclusion

The isolates of *H. pylori* strains from TT are mostly carriers of the *vac*A s2/m1, which is implicated in most of the associated infections. There are great similarities between local strains based on the PCR and DNA sequencing patterns to those seen in the USA, India, and Indonesia. Therefore, factors contributing to the emergence of s2/m1 will be more prevalent and need to be further investigated.

## Funding

Lemar Wayne Blake further extends gratitude to the School of Veterinary Medicine, Faculty of Medical Sciences, The University of the West Indies, St. Augustine, for assisting with funds for genomic sequencing. The authors declare that there are no conflicts of interest.

## Ethical approval

The ethical approval was obtained from the University of the West Indies St. Augustine Campus, Trinidad and Tobago, and the Private Gastroscopy clinic before the collection and processing of patient samples. Written or verbal consents of the patient were also obtained prior to each investigation which was to subject the patients to gastroscopy procedures for sample collection.

## Declarations of competing interest

The authors have no competing interests to declare.
